# Embodiment in Virtual Reality Intensifies Emotional Responses to Virtual Stimuli

**DOI:** 10.3389/fpsyg.2021.674179

**Published:** 2021-09-06

**Authors:** Dominik Gall, Daniel Roth, Jan-Philipp Stauffert, Julian Zarges, Marc Erich Latoschik

**Affiliations:** Human-Computer Interaction, Institute of Computer Science, Universtiy of Würzburg, Würzburg, Germany

**Keywords:** embodiment, virtual body ownership, avatars, agency, immersive interfaces, human-computer interaction, affective computing, emotions

## Abstract

Modulating emotional responses to virtual stimuli is a fundamental goal of many immersive interactive applications. In this study, we leverage the illusion of illusory embodiment and show that owning a virtual body provides means to modulate emotional responses. In a single-factor repeated-measures experiment, we manipulated the degree of illusory embodiment and assessed the emotional responses to virtual stimuli. We presented emotional stimuli in the same environment as the virtual body. Participants experienced higher arousal, dominance, and more intense valence in the high embodiment condition compared to the low embodiment condition. The illusion of embodiment thus intensifies the emotional processing of the virtual environment. This result suggests that artificial bodies can increase the effectiveness of immersive applications psychotherapy, entertainment, computer-mediated social interactions, or health applications.

## 1. Introduction

Human-computer interfaces like virtual reality systems can display immersive virtual environments where participants can feel fully immersed and present in the virtual environment (Slater, 2018). Moreover, through virtual reality systems, it is possible to induce the sense of embodiment toward virtual bodies (Slater et al., 2008). Users not only see a virtual body in place of their own, but they can also develop the sensation that the virtual body parts are parts of their own body. Botvinick and Cohen (1998) first reported the observation that we can induce the illusion that an unanimated object is part of one's own body through multisensory correlations such as visuotactile stimulation: the authors sat participants in front of a rubber hand aligned to their real hidden one. Then, they applied synchronous tactile stimulation with two paintbrushes to both hands. Participants reported experiencing the illusion that the rubber hand, to some degree, was their natural hand. People can experience a similar illusion in virtual reality setups, where visuotactile stimulation can be applied simultaneously to a virtual body representation and the natural body. We can describe this sensation to own an artificial or virtual body as the illusion of virtual body ownership. This illusion is part of a more general experience, the sense of embodiment toward an artificial body. Following the working definition of Kilteni et al. (2012), embodiment describes the sensation that our self is located inside a virtual body; we control this body and that this body belongs to us. Hence, embodiment consists of three subcomponents, the self of presence (or self-location in the original paper), the sense of agency, and the sense of body ownership. Presence or self-location refers to the sensation of physically being at a virtual place while knowing that one is not there (Akin et al., 1983; Skarbez et al., 2017). Agency describes the sensation of controlling a virtual body with one's own will. And finally, body ownership describes the sensation that a body is the source of one's sensation (see e.g., Braun et al., 2018 for a discussion of the concepts virtual body ownership and agency).

Previous investigations demonstrated the applicability of the sensation of embodiment in different virtual reality applications, such as health (Ramakonar et al., 2011; Martini, 2016; Matamala-Gomez et al., 2019, 2021) and entertainment (Riva et al., 2016; Slater and Sanchez-Vives, 2016; Xu et al., 2020). However, we know little about the mechanisms of how virtual embodiment affects the effectiveness of such applications.

The illusion of virtual embodiment and its subcomponents can arise in many different circumstances. The visual impression to have a virtual body often suffices to induce the sense of ownership (e.g., Vogeley et al., 2004; Kokkinara et al., 2016; Burin et al., 2020). Additionally, multisensory feedback provides means to manipulate the strength of the illusion. In the original rubber hand experiment, participants, for example, saw a pencil stroking a rubber hand while feeling a simultaneous stroke on their own hand (Botvinick and Cohen, 1998). The rubber hand was only slightly displaced to the real hand. Since the integration of different sensory information is based on imprecise estimation processes, this process can err, leading to such an illusion (Ernst and Banks, 2002). Several factors can contribute to the induction of virtual embodiment (see Kilteni et al., 2015 for a review): First, the shape, texture, and position of the virtual body seem sufficiently plausible. Second, the virtual body appears in the same place as their real body. Third, the virtual body gets touched in the same place and time as the real body. Fourth, the virtual body moves synchronously to the real body.

What we know is that the illusion of virtual embodiment affects not only the perception of one's body but has an impact on cognitive processes: virtual body ownership, as a subcomponent of embodiment can influence our spatial perception, for example the perceived location of the real body part (Botvinick and Cohen, 1998; Lenggenhager et al., 2007; Sanchez-Vives et al., 2010), once spatial orientation (Preuss et al., 2018), or the perceived size and distance of virtual objects (Van Der Hoort et al., 2011; Banakou et al., 2013; Linkenauger et al., 2013; Van der Hoort and Ehrsson, 2014; Van Der Hoort and Ehrsson, 2016; Jung et al., 2018). Virtual body ownership can affect social perception. It influences how we identify with social groups. It can affect the attitudes toward the social group to which the virtual body belongs (Farmer et al., 2012, 2014; Banakou et al., 2013; Fini et al., 2013; Maister et al., 2013; Peck et al., 2013). Moreover, virtual body ownership can modualte pain perception (Martini et al., 2013, 2014, 2015; Osumi et al., 2014; Giummarra et al., 2015; Nierula et al., 2017; Matamala-Gomez et al., 2018, 2019, 2020b), the perceived body weight (Serino et al., 2016; Chirico et al., 2019; Wolf et al., 2020), motor performance (Matamala-Gomez et al., 2020a) or memory processes (Tacikowski et al., 2020b). Virtual body ownership also affects responses to physical threat stimuli delivered toward to the virtual body (Ehrsson, 2007; Ehrsson et al., 2007; Petkova and Ehrsson, 2008; Gentile et al., 2013). Previous studies also showed, that virtual body ownership affect threat-evoked skin conductance responses (Ehrsson, 2007; Petkova and Ehrsson, 2008, 2009; Gentile et al., 2013; Guterstam et al., 2015; Tacikowski et al., 2020b) and threat evoked fMRI activations in areas related to fear and pain anticipation during rubber hand illusion (Ehrsson et al., 2007; Gentile et al., 2013). Body ownership changes in full-body illusion paradigms are also related to feelings of body satisfaction (Preston and Ehrsson, 2014, 2016) and feelings of femininity or masculinity (Tacikowski et al., 2020a).

These findings suggest that virtual embodiment might have a general effect on information processing processes. General moderators for information processing are emotional reactions to virtual stimuli. Emotional reactions play an important role in immersive systems, for example, in health applications like virtual exposure therapy or virtual social interactions (Riva et al., 2007). Emotions organize motivation, attention, memory, performance, and decision-making Oatley et al. (2006). There are specific findings that virtual embodiment modulates anxiety and arousal for stimuli that directly threaten the virtual body (Romano et al., 2014; Guterstam et al., 2015; Argelaguet et al., 2016; Chen et al., 2017). However, we do not know the impact of virtual embodiment on the intensity of general emotional reactions to virtual stimuli to date.

We present results from a study that supports the hypothesis that virtual embodiment intensifies emotional reactions to virtual stimuli. However, we cannot determine whether the subcomponents, ownership, agency, or presence drive the effect. The goal of our study was to investigate if virtual embodiment modulates the emotional responses to the virtual environment. We use the three-dimensional conceptualization of emotions as valence, arousal, and dominance (Osgood, 1952; Russell and Mehrabian, 1977). We designed one experimental condition that aimed to induce low levels of virtual embodiment and one condition that aimed to induce high virtual embodiment levels. We then compared emotional reactions to visual stimuli within the environment. We used subjective rating scales to assess these emotional reactions. Our results suggest that virtual embodiment increases the intensity of emotional responses to general virtual stimuli. We conclude that virtual embodiment might play an active role in organizing emotional responses to virtual stimuli. This finding encourages the introduction of virtual bodies for immersive scenarios that aim to intensify emotional responses in users. Applications may lie in entertainment, computer-mediated social interactions, or health applications.

## 2. Methods

### 2.1. Study Design and Participants

The experiment followed a single-factor repeated-measures design with two-factor levels. We manipulated the factor temporal synchrony of visuotactile and passive visuomotor stimulation (synchronous vs. asynchronous). We assumed that participants would experience higher virtual embodiment in the synchronous condition. Participants completed the two conditions in balanced, randomized order.

Undergraduate female students from the University of Würzburg volunteered to participate in the experiment. We only included female participants because we selected the stimuli according to female norm values (Lang, 2005). The sample consisted of *N* = 21 women, with age ranging from 19 to 30 years (*M* = 21.62, *SD* = 2.80). All participants provided written informed consent before participation. They received course credit for participation. All reported normal or corrected-to-normal vision and the absence of motor impairments. Participants were naive regarding the hypotheses of the experiment. The institutional ethics committee Human-Computer-Media approved this study on April 10, 2018.

### 2.2. Apparatus

**Figure 1:**
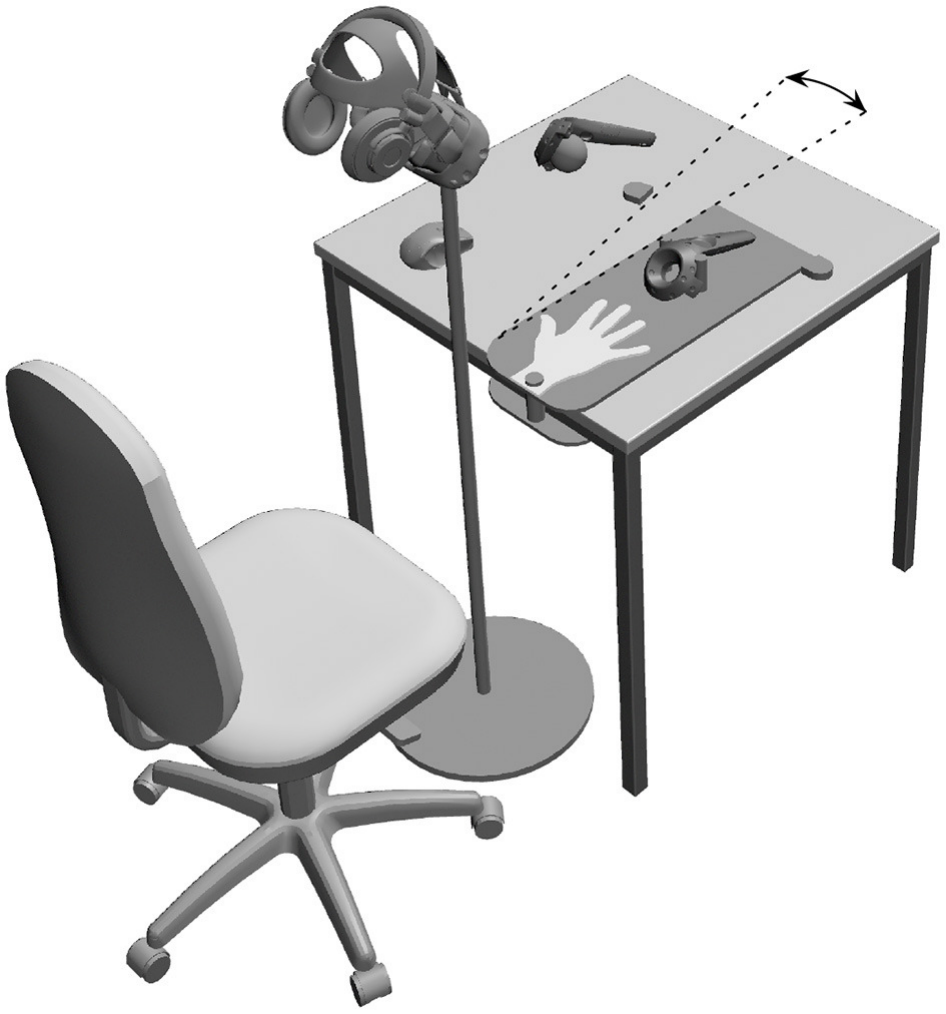
Apparatus. We presented visual stimuli in a immovable head-mounted display. Participants placed their right hands on a hand rest. The experimenter delivered visuotactile stimulation with a styrofoam ball attached to a position sensor. The experimenter delivered visuomotor stimulation by rotating the hand rest. A sensor tracked the position of the hand rest. We presented Brownian noise in headphones.

During the experiment, the right hand of participants rested on a hand rest. The hand rest defined static positions for each finger. The examiner could rotate the hand rest around the wrist by 13 degrees without touching the participant. A position sensor (HTC Vive Controller) tracked the rotation angle. The examiner could stroke the hand of the participant with a styrofoam ball. The styrofoam ball had a diameter of 5 cm. A position sensor (HTC Vive Controller) tracked the position of the styrofoam ball. Participants looked through an immovable head-mounted display (HTC Vive Headset). The static field of view comprised the right hand at a distance of 35 cm. An Intel i7 4.00 GHz, 16 GB RAM computer with an NVIDIA GeForce GTX 1080 Ti graphics card rendered stereoscopic images at 90 Hz. We implemented sensor data integration, visualization of the virtual environment, and response registration in the Epic Games Unreal Engine 4.17. We presented auditive stimuli with a headphone (Superlux HD330). Figure 1 illustrates the apparatus.

### 2.3. Stimuli

Participants saw a virtual representation of their right hand. The virtual hand appeared in the same position as the real hand. During visuotactile stimulation, participants also saw a virtual representation of the styrofoam ball. In the synchronous stimulation condition, the virtual styrofoam ball appeared in the same position as the real styrofoam ball. In the synchronous stimulation condition, the virtual hand rotated analogous to the real hand. In the asynchronous stimulation condition, we delayed the movements of the virtual styrofoam ball and the virtual hand by 5 s. To neutralize noises of the tactile or motoric stimulation, we presented Brownian auditive noise through the headphones at 50 dB during the visuotactile and visuomotor stimulation. A calibration measurement of the system showed that the delay in the synchronous conditions was below 40 ms. The threshold for detecting visuomotor delays is above 100 ms (Franck et al., 2001; Shimada et al., 2009, 2010).

**Figure 2:**
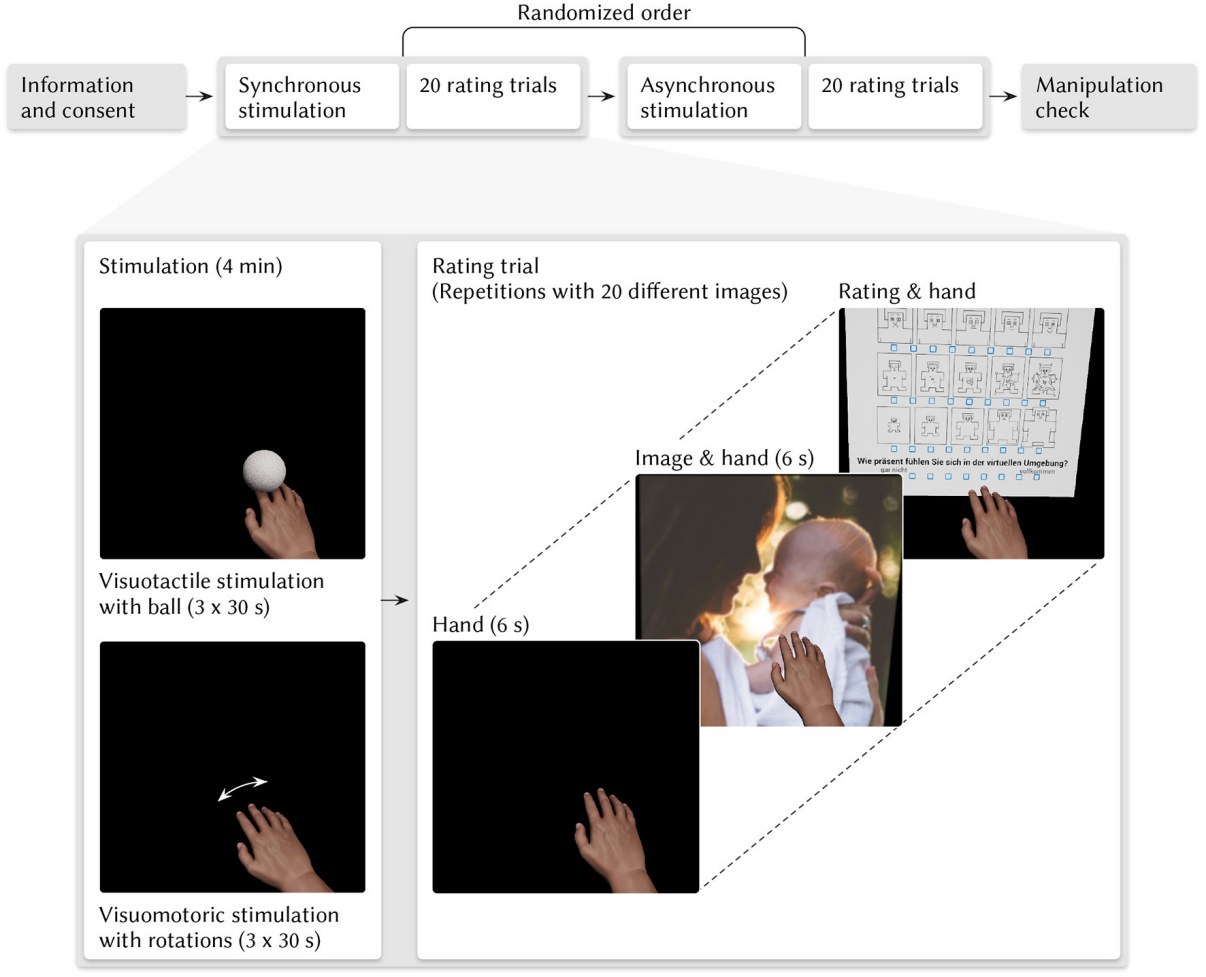
Procedure and stimuli. Participants completed the synchronous and the asynchronous condition in a balanced randomized order. In each condition, participants received visuomotor and visuotactile stimulation for 4 min. We stroked the hands of participants with a ball and rotated their hands. In the synchronous condition, the hand and the virtual ball moved synchronously to the real hand and real ball. In the asynchronous condition, the virtual objects moved 5 s delayed. Afterward, participants completed 20 trials in each condition. In each trial, participants saw the virtual hand for 6 s, then an image with the virtual hand for 6 s. Afterward, they completed self-assessment manikin and presence ratings. After completing both conditions, participants answered a questionnaire to assess if the manipulation worked. Placeholder image stimulus by Julie Johnson on Unsplash.

In each condition, we presented 20 pictures from the international affective picture system (Lang, 2005). The pictures appeared on a frontal plane behind the virtual hand. The virtual index finger pointed to the center of the image. The virtual hand covered a lower right fraction of the image. So participants still saw the virtual hand when they looked at the image. The 20 images consisted of 10 images with low and ten images with high valence female norm values. On average, the female norm arousal values of both groups were approximately equal. For the positive image group, female norm values on a Likert scale from 1 (*negative/low*) to 9 (*positive/high*) were (Lang, 2005): valence *M* = 7.00, *SD* = 0.62; arousal *M* = 6.79, *SD* = 0.28; dominance 5.38, *SD* = 0.34. For the negative image group, female norm values on a Likert scale from 1 (*negative/low*) to 9 (*positive/high*) were: valence *M* = 2.47, *SD* = 0.43; arousal *M* = 6.63, *SD* = 0.43; dominance 3.10, *SD* = 0.71 (Lang, 2005). We selected the images with maximal norm values for arousal and valence (very high or very low valence) while minimizing the possibility to choose pictures that might be ethically controversial. The positive valent image group consisted of the following images (international affective picture system number with a description in brackets): 4,525 (attractive male), 4,668 (erotic couple), 4,698 (erotic couple), 5,621 (skydivers), 5,629 (hiker), 8,001 (basketball player), 8,158 (hiker), 8,179 (bungee jumper), 8,180 (cliff divers), and 8,185 (skydivers). The negative valent image group consisted of the following images (international affective picture system number and description in brackets): 9,623 (fire), 9,600 (ship), 7,380 (roach on pizza), 6250,1 (aimed gun), 6,220 (boys with guns), 2,811 (gun), 1,932 (shark), 1,271 (roaches), 1,201 (spider), and 1,052 (snake). Figure 2 depicts the virtual stimuli.

### 2.4. Procedure

Participants sat in front of a table. They placed their right hand on a hand rest and wore an immovable head-mounted display with headphones. In the synchronous and asynchronous conditions, participants continuously saw a virtual representation of their right hand.

At the beginning of each condition, the experimenter delivered visual-tactile and passive visuomotor stimulation. We alternated between visual-tactile and passive visuomotor stimulation every 30 s for a total of 4 min. The experimenter manually delivered visual-tactile stimulation by stroking the index and middle finger from the fingertips to the back of the hand with the styrofoam ball. In the synchronous condition, the virtual styrofoam ball synchronously strokes the virtual hand. In the asynchronous condition, the virtual styrofoam ball moved 5 s delayed. The experimenter manually delivered passive visuomotor stimulation by rotating the hand rest around 13 degrees. In the synchronous condition, the virtual hand rotated synchronously. In the asynchronous condition, the virtual hand rotated 5 s delayed.

After the stimulation period, participants completed 20 rating trials. In each trial, participants saw the virtual representation of their right hand for 6 s. Then, participants saw an image from the international affective picture system on a frontal plane behind the virtual hand for 6 s. Afterward, participants self-assessed valence, arousal, and dominance on self-assessment manikin scales and a one-item presence scale. The scales appeared behind the virtual hand. Participants used a mouse in their left hand to select answers and to continue to the next trial.

Afterward, we conducted mid-immersion one-item virtual embodiment and agency ratings with the headphones. Between the two conditions, participants rested for 5 min. Figure 2 illustrates the procedure.

### 2.5. Measures

After each trial, participants completed a questionnaire that consisted of self-assessment manikin scales and a one-item presence scale. The questionnaire appeared on a frontal plane behind the virtual hand. The virtual hand did not cover parts of the questionnaire. We used the self-assessment manikin scales (Bradley and Lang, 1994) to assess emotional responses in each trial. Self-assessment manikin scales allow non-verbal pictorial assessment of self-reported affective experience immediately after stimulus presentation. Self-assessment manikin scales assessed norm values for international affective picture system images (Lang, 2005). We used self-assessment manikin scales with five pictures, labeled with a 9-point Likert scale from 1 (*low*/*negative*) to 9 (*high*/*positive*). This measure assumes the conceptualization of emotion as three independent dimensional bipolar factors: valence, arousal, and dominance (Osgood, 1952; Russell and Mehrabian, 1977). Valence conceptualizes approach or avoidance tendencies. Arousal conceptualizes the perceived level of physiological activity. Dominance conceptualizes the perceived level of control. Before the experiment, we described the self-assessment manikin scales to the participants, as proposed by Lang et al. (1999) Dimensional self-reports about affective experiences made directly after an emotion eliciting event have reasonable validity (Mauss and Robinson, 2009). Validity and reliability of the self-assessment manikin scales are reasonable (Bradley and Lang, 1994). Dominance is the least sensitive scale among the three and correlates positively with valence (Russell, 1979; Bradley and Lang, 1994; Warriner et al., 2013).

Furthermore, we used a visual one-item mid-immersion presence scale to assess self-reported presence in each trial, as proposed in Bouchard et al. (2008). Participants answered the following question on a scale of 1 (*not at all*) to 9 (*totally*): “To what extent do you feel present in the virtual environment?” There is evidence that brief one-item presence measures during immersion are more sensitive to the subjective feeling of presence than post-immersive questionnaires (Freeman et al., 1999; Slater, 1999; Bouchard et al., 2008). Hendrix and Barfield (1995) confirmed the reliability of a similar presence rating. Others showed the ability of this and similar measures to detect treatment effects (Hoffman et al., 2006; Bouchard et al., 2008; Kober et al., 2012) gives preliminary evidence of its validity.

At the end of each condition, we assessed virtual embodiment and a proxy measure for agency while participants still saw the virtual hand. We presented auditive one-item mid-immersion questions through the headphones. Participants answered the following questions out loud on a scale of 0 (*not at all*) to 10 (*totally*): “To what extent do you have the feeling as if the virtual body is your body?” (virtual embodiment, adapted from Kalckert and Ehrsson, 2012) and “To what extent do you have the feeling that the virtual body moves just like you want it to as if it is obeying your will?” (proxy measure for agency, however, no active movement was involved adapted from Kalckert and Ehrsson, 2012).

### 2.6. Statistical Analysis

We used linear mixed models to compare valence, arousal, dominance, and presence ratings between the synchronous and the asynchronous stimulation condition. We used lme4 (Bates et al., 2015) in R 3.5.1. (R Core Team, 2018) to model participants and stimuli as additive random effects to account for their interdependence in the repeated measurements design (e.g., arousal ~ (1|participant) + (1|stimulus) + condition). We used lmerTest (Kuznetsova et al., 2017) to calculate *p*-values. We compared valence ratings separately for stimuli with high (i.e., positive) and low (i.e., negative) norm valence. Positive and negative valent stimuli modulate the intensity of valence ratings in inverted directions. To assert if our manipulation worked, we used two-tailed paired *t*-tests to compare virtual body ownership and agency ratings between the synchronous and asynchronous stimulation conditions. To achieve a global alpha level of 5%, we Bonferroni-Holm adjusted the significance thresholds of the tests for multiple comparisons. We report Cohen's *d* as a measure of effect size. Data and code for all analyses are available at https://osf.io/quysv/.

## 3. Results

We examined whether virtual embodiment increases emotional responses to virtual stimuli. Therefore, we manipulated the degree of virtual embodiment and assessed responses to emotional stimuli. We used subjective rating scales for valence, arousal, and dominance to assess the intensity of emotional responses. To check if the experimental manipulation works, we assessed virtual body ownership and a proxy measure for agency at the end of each condition, and we assessed presence after each stimulus. We collected all data without technical problems and hence included the subjective ratings of all 21 participants in the repeated measures analysis.

We presented stimuli with high norm arousal and dominance. In the synchronous stimulation condition participants reported significantly higher arousal (*p* = 0.002, *d* = 0.18), and dominance (*p* = 0.003, *d* = 0.19) compared to the asynchronous stimulation condition. We presented stimuli with positive and negative valence. Positive valence translates to high ratings and negative valence translates to low ratings. Since positive and negative valence ratings nullify if analyzed together, we analyzed valence ratings separately for stimuli with high (i.e., positive) and low (i.e., negative) norm valence. For positive valent stimuli, participants reported significantly higher valence ratings (*p* < 0.001, *d* = 0.27) in the synchronous compared to the asynchronous stimulation condition. For negative valent stimuli, participants did not report a significant difference in the synchronous compared to the asynchronous stimulation condition (*p* = 0.877). Table 1 lists the detailed results of these mean comparisons.

**Table 1:** Mean comparisons.

**Rating scale**	**Synchronous** **mean (SD)**	**Asynchronous** **mean (SD)**	**Difference**	**95% CI**	***t***	***p***	***d***
Valence (positive stimuli)	5.99 (1.31)	5.66 (1.49)	0.33	[0.14, 0.51]	3.50	^***^	0.27
Valence (negative stimuli)	3.98 (1.75)	3.99 (1.57)	–0.01	[–0.22, 0.19]	–0.14	0.891	
Arousal	4.83 (1.72)	4.54 (1.73)	0.29	[0.10, 0.47]	3.05	0.002	0.18
Dominance	4.99 (1.74)	4.70 (1.83)	0.28	[0.10, 0.47]	3.02	0.003	0.19
Presence	4.30 (1.99)	3.30 (1.81)	1.01	[0.84, 1.18]	11.54	^***^	0.64

*Summary of mean comparisons of valence (separated by positive and negative stimuli), arousal, dominance, and presence ratings*.

*Ratings range from 1 (low/negative/not at all) to 9 (high/positive/totally). CI, confidence interval; SD, standard deviation*.

*** *p < 0.001*.

**Figure 3:**
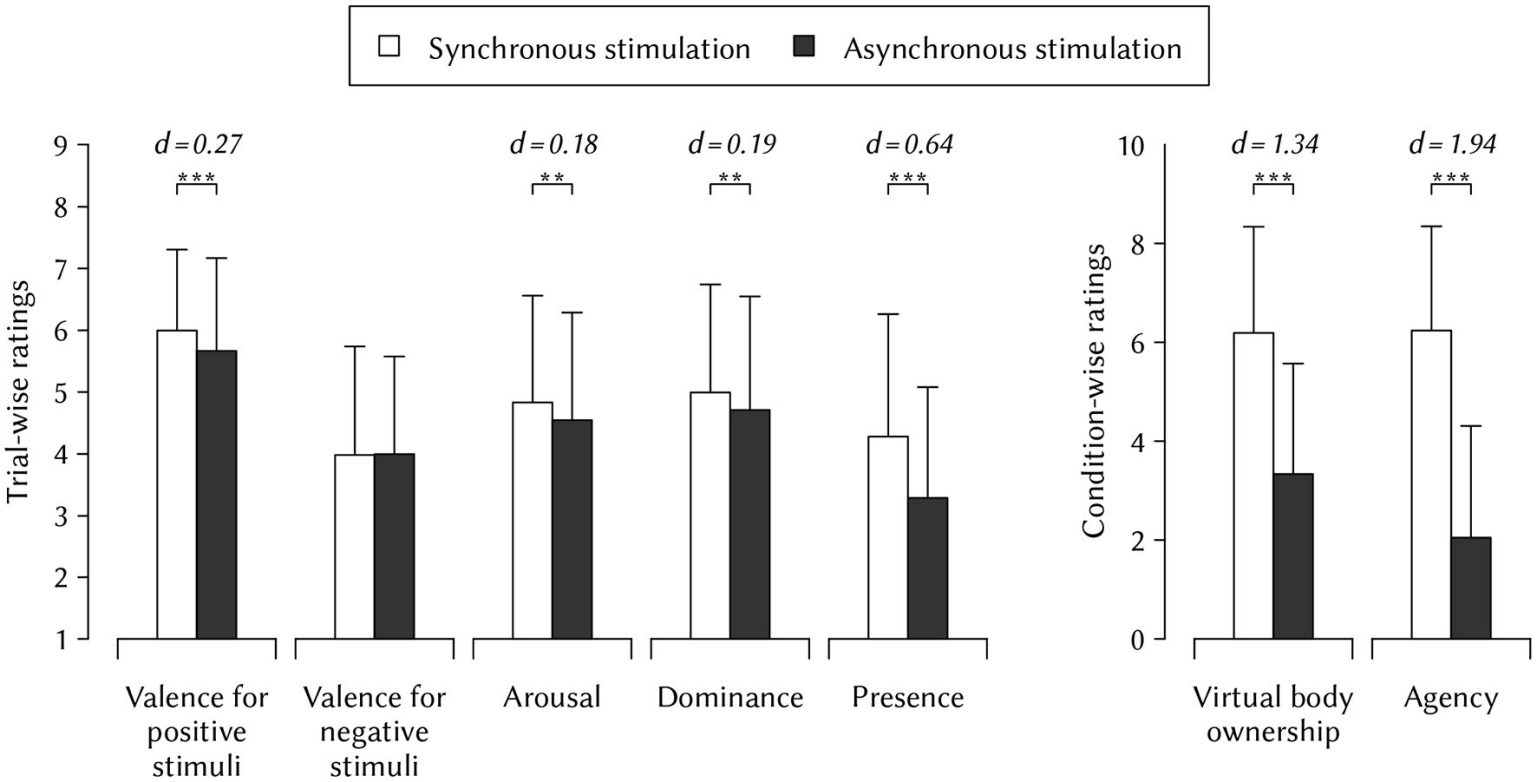
Results. Mean (+ SD) of trial-wise self-assessment manikin and presence ratings, as well as condition-wise questionnaire ratings. Participants reported significantly higher valence for positive stimuli, arousal, dominance, and presence when they receive synchronous compared to asynchronous stimulation. The manipulation check revealed significantly higher virtual embodiment and agency after synchronous compared to asynchronous stimulation. The *p*-values indicate significant results of Bonferroni-Holm adjusted mean comparisons. Cohen's *d* values indicate effect sizes. Self-assessment manikin ratings range from 1 (*low*/*negative*) to 9 (*high*/*positive*). Questionnaire ratings range from 0 (*not at all*) to 10 (*totally*). Abbreviations: **p < 0.01, ***p < 0.001.

We checked if the intended manipulation of virtual embodiment worked. To this end, we assessed virtual body ownership and agency in each condition after participants completed the trials, and we assessed presence in each trial. In the synchronous stimulation condition participants reported significantly higher virtual body ownership [*t*_(20)_ = 6.15, *p* < 0.001, *d* = 1.34] and agency [*t*_(20)_ = 8.99, *p* < 0.001, *d* = 1.96] compared to the asynchronous stimulation condition. Paricipants also reported higher presence ratings in the synchronous (*M* = 4.30, *SD* = 1.99) compared to the asynchronous condition (*M* = 3.30, *SD* = 1.81, *p* < 0.001, *d* = 0.64). Figure 3 depicts the mean comparisons of the virtual body ownership and agency.

## 4. Discussion

### 4.1. Main Findings

Numerous studies have demonstrated the potential of virtual embodiment in shaping cognitive processes. But despite the profound impact of virtual embodiment on the sense of self, there is a lack of understanding about how virtual embodiment affects fundamental information processing in human-computer interaction. This lack has impeded leveraging the effects of virtual embodiment to engage users of immersive systems. Our study now suggests that virtual embodiment affects a fundamental quality of information processing: the emotional responses to stimuli. The induction of emotions is central for many applications in human-computer interaction, from virtual therapy to entertainment (Brave and Nass, 2003). We hypothesized that the sensation of virtual embodiment changes emotional reactions to stimuli in the virtual environment and obtained experimental support for this hypothesis. In our experiment, we manipulated virtual embodiment and assessed the emotional responses to stimuli in the virtual environment. Participants reported higher emotional responses regarding arousal, dominance, and valence in the high embodiment condition compared to the low embodiment condition. A manipulation check confirmed that the experimental setup induced virtual embodiment as intended. We conclude that the illusion of virtual embodiment modulates the emotional responses to the virtual environment and hence provides means to leverage the effectiveness of immersive virtual reality systems. However, our results do not allow us to conclude which subcomponents of embodiment drive the effect. Our manipulation yielded changes in all three subcomponents of embodiment: virtual body ownership, agency, and presence (Kilteni et al., 2012).

To induce a sense of agency, the experimenter rotated the hand for a total of 2 min in each condition. The rotation movement of the hand rest was uniform and determined only by the experimenter. Hence participants could anticipate the action but could not actively control the virtual hand. Even though this manipulation did not include motor intention, participants reported a different degree of how the virtual hand obeyed their will. We assume that the uniform movement of the hand rest induced a voluntary action that mimicked the expected hand rotation. We decided only to use passive motor movements to increase standardization between participants, however to the cost of decreasing the validity of the measure. However, we can only use the term agency with caution since voluntary actions can be considered a prerequisite for experiencing agency (Gallagher, 2000).

Participants reported slightly higher arousal, dominance, and more positive valence to positive valent stimuli. We assume that higher arousal might since feeling embodied within an environment might increase the relevance of the surrounding object to oneself. For example, threatening objects might appear more dangerous if one imagines being in that place (Zhang and Hommel, 2016). Valence describes as the degree of pleasant or unpleasantness one perceives (Russell, 1979). We hypothesize that more intense valence ratings for positive stimuli reflect increased engagement in the virtual surrounding of one feels embodied in that world. We could not see this association for negative valent stimuli. For negative valent stimuli, this moderation effect of embodiment might be overshadowed by a more intense reaction to aversive stimuli. The result might raise the hypothesis that the effect of virtual embodiment might be different for positive and negative valent stimuli. Dominance ratings might relate to a feeling of being in control. Feeling embodied in a virtual body might increase the sensation of being able to control the surrounding environment, hence exerting more dominance.

The hypothesis that virtual embodiment affects emotions might inform theories about emotional processes. On the one hand, our findings support theories that assume that emotions express the relevance of stimuli to personal goals (see Nelissen et al., 2007 for a review). We hypothesize that having a body in an environment increases the relevance of entities in this environment since they can impact our bodies. Therefore, the presence of a body in a particular environment might increase the relevance of that environment to one's goals. On the other hand, our findings could raise the hypothesis that there might also be an inverse association of embodiment and emotional responses: That reduced embodiment might reduce emotional responses. This hypothesis is consistent with the theory of depersonalization, a body function that decreases intense emotional reactions in highly threatening situations (Simeon et al., 2000; Sierra and David, 2011; Dewe et al., 2016, 2018).

### 4.2. Limitations

Our method does not allow to identify which subcomponent of embodiment drives the observed effect (virtual body ownership, agency, or presence), since our manipulation affected all three, at least to some degree. It might also be possible that the sensation of presence mediates the effect. There is some evidence that presence relates to emotional reactions. However, the results are controversial (Schuemie et al., 2001). Some studies found correlations between presence and fear reactions, but only if arousal was sufficiently high (see Diemer et al., 2015 for a review). Presence, however, does not relate to outcomes of virtual exposure therapy (Diemer et al., 2015). Some studies indicate a causal influence of emotions on presence (Bouchard et al., 2008; Gorini et al., 2011; Gromer et al., 2019).

Our results suggest that the effects of virtual embodiment on emotional responses to virtual stimuli exist but that it might be small. Small effect sizes in our study can be due to three reasons: first, the intensity of the displayed stimuli, in general, was low. Due to ethical reasons, we selected images that do not induce intense emotional reactions. The general, moderate arousal ratings reflect this selection. Second, the stimuli were two-dimensional image stimuli within the virtual environment. Hence the presented stimuli induced a second layer of virtuality: virtual stimuli within a virtual world. This abstraction might loosen the association of the stimuli to the virtual body and hence their emotional relevance. Third, we presented stimuli out of context, stimuli without relation to the participants' personal goals. Since emotions provide information about goal attainment states, we expect only a moderate reaction to such out-of-context stimuli. However, our data imply a causal relationship between virtual embodiment and emotional responses despite these three issues. This finding underlines this relationship's potential in settings with virtual stimuli that have high relevance for users.

In our study, we used a well-established but still reductionist concept to measure emotional reactions: We used a three-dimensional, continuous model to operationalize emotional reactions, and we assessed this model with retrospective self-reports. We assume that this model may only capture a simplistic image of the actual emotional responses of participants. However, our results suggest that even such a simple model reveals a causal effect of virtual embodiment on emotional responses. Further studies might qualify and differentiate this effect.

We used only a virtual hand to represent a virtual body to reduce visual inconsistencies that might break the illusion of being embodied Kilteni et al. (2015). However, a full-body illusion might yield stronger effects. It is also possible that the effect of embodiment faded away during the exposure to the virtual stimuli. Our study by design did not allow to detect such an effect over time. However, we did not find meaningful differences when comparing the first and the second half of the trials in an exploratory analysis (see analysis in https://osf.io/quysv/).

A conservative power simulation of the model reveals a power of 50%. Hence the study might be underpowered, increasing the probability that the findings reported here are false positives. Hence we interpret the results with caution, and treat them in an exploratory manner and encourage replication of our findings.

We only used three items to assess virtual embodiment, one item for body ownership, agency, and presence. We also did not use physiological measures to operationalize a sensation of ownership. Hence, more elaborated measures for the discussed constructs might increase the external validity in future studies. So we consider our results as preliminary findings that require replication with more specific operationalizations of the subconstructs of embodiment. We also did not include control statements to control for random answers or phantom sensations since we tried to keep the trials as short as possible and assume that such effects would be equally distributed across the two conditions due to the counter-balanced design.

We can not exclude that the results of our study are confounded with other cognitive processes that may have varied systematically between the two conditions. For example, participants might feel more distracted or irritated in the asynchronous condition leading to a lesser engagement and hence reduce emotional engagement. However, we assume that an aversive reaction to the asynchronous stimulation would increase arousal levels (Craig, 1968) in the asynchronous condition compared to the synchronous condition, and hence be contrary to the observed direction.

### 4.3. Implications

Despite technological advancements in immersive systems in health, entertainment, and computer-mediated collaborative work, virtual bodies to date only play a minor role in customer applications. For example, many studies investigate virtual reality for the treatment of psychopathologies. Notably, virtual reality exposure therapy reduces anxiety in phobic disorders (Freeman et al., 2017), where a higher activation of emotions relates to positive treatment effects (e.g., Greenberg and Pascual-Leone, 2006; Craske et al., 2014). However, previous clinical studies that use virtual stimuli mostly did not present virtual bodies (Freeman et al., 2017). In line with Braun et al. (2018), our findings encourage the development of technologies that facilitate a robust induction of virtual embodiment. Such developments would allow inducing virtual embodiment and hence intensify the emotional response to therapeutic stimuli. Then virtual embodiment, in turn, might enhance treatment outcomes of virtual reality interventions.

### 4.4. Conclusion

We provide experimental support for the hypothesis that virtual embodiment intensifies emotional response to virtual stimuli. The increase in emotional responses is essential for many human-computer interaction applications such as virtual psychotherapy, entertainment, or computer-mediated cooperative work. Our results encourage the development of technologies that allow leveraging virtual embodiment to increase the effectiveness of virtual reality systems.

## Data Availability Statement

Data and code for all analyses are available at https://osf.io/quysv/.

## Ethics Statement

The studies involving human participants were reviewed and approved by Universtiy of Würzburg, Human-Computer-Media. The participants provided their written informed consent to participate in this study.

## Author Contributions

ML and DG designed and planned the study. DR and J-PS helped to design the study supported the creation of the apparatus and software. JZ collected the data and contributed to the creation of the apparatus and software. DG analyzed the data and wrote the first draft of the manuscript. ML supervised the data collection and data analysis. All authors contributed to the final manuscript.

## Conflict of Interest

The authors declare that this study received resources (namely the 3D model of the virtual hand) from Gotoxy AV-Media GbR, Nuremberg, Germany. The company was not involved in the study design, collection, analysis, interpretation of data, the writing of this article or the decision to submit it for publication.

## Publisher's Note

All claims expressed in this article are solely those of the authors and do not necessarily represent those of their affiliated organizations, or those of the publisher, the editors and the reviewers. Any product that may be evaluated in this article, or claim that may be made by its manufacturer, is not guaranteed or endorsed by the publisher.
